# Functional dissection of the alphavirus capsid protease: sequence requirements for activity

**DOI:** 10.1186/1743-422X-7-327

**Published:** 2010-11-18

**Authors:** Saijo Thomas, Jagdish Rai, Lijo John, Stephan Günther, Christian Drosten, Brigitte M Pützer, Stephan Schaefer

**Affiliations:** 1 Department of Vectorology and Experimental Gene Therapy, Biomedical Research Center, University of Rostock, D-18057 Rostock, Germany; 2 Beckman Institute, Urbana, Illinois 61801, USA; 3 Department of Virology, Bernhard Nocht Institute for Tropical Medicine, D-20359 Hamburg, Germany; 4 Institute of Virology, University of Bonn Medical Center, D-53127 Bonn, Germany

## Abstract

**Background:**

The alphavirus capsid is multifunctional and plays a key role in the viral life cycle. The nucleocapsid domain is released by the self-cleavage activity of the serine protease domain within the capsid. All alphaviruses analyzed to date show this autocatalytic cleavage. Here we have analyzed the sequence requirements for the cleavage activity of Chikungunya virus capsid protease of genus alphavirus.

**Results:**

Amongst alphaviruses, the C-terminal amino acid tryptophan (W261) is conserved and found to be important for the cleavage. Mutating tryptophan to alanine (W261A) completely inactivated the protease. Other amino acids near W261 were not having any effect on the activity of this protease. However, serine protease inhibitor AEBSF did not inhibit the activity. Through error-prone PCR we found that isoleucine 227 is important for the effective activity. The loss of activity was analyzed further by molecular modelling and comparison of WT and mutant structures. It was found that lysine introduced at position 227 is spatially very close to the catalytic triad and may disrupt electrostatic interactions in the catalytic site and thus inactivate the enzyme. We are also examining other sequence requirements for this protease activity.

**Conclusions:**

We analyzed various amino acid sequence requirements for the activity of ChikV capsid protease and found that amino acids outside the catalytic triads are important for the activity.

## Background

Alpha viruses are spherical, enveloped, positive-sense ssRNA viruses responsible for a considerable number of human and animal diseases. Alphavirus members include Chikungunya virus (ChikV), Sindbis virus, Semliki Forest virus (SFV), the Western, Eastern and Venezuelan equine encephalitis viruses, and the Ross River virus. Some alphaviruses can cause arthritic diseases and encephalitis in humans and animals and continue to be a worldwide threat. The viruses are transmitted by blood-sucking arthropods, and replicate in both, arthropod and vertebrate hosts [1]. Among alphaviruses, ChikV is considered a re-emerging virus in the family of Togaviridae. ChikV infection produces an illness in humans that is characterized by fever, headache, nausea, vomiting, myalgia, rash, arthralgia, encephalitis, and lethal hepatitis [2].

The alphaviral single-stranded RNA genome is packaged into a nucleocapsid made up of 240 copies of the basic capsid protein [3]. The nucleocapsid is surrounded by a lipid bilayer containing two integral glycosylated membrane proteins (E2 and E1) and a small peripheral protein E3. The alphavirus capsid is multifunctional and plays a key role in the viral life cycle. It is made up of two domains, the unstructured, RNA binding N-terminal segment (residues 1-118) and the C-terminal globular protease domain (residues 119-267) [4]. The serine protease domain consists of a β-barrel motif with a typical serine-histidine-aspartic acid catalytic triad with the active site interspersed between two subdomains [5,6].

The first cleavage event in SFV is executed by the serine protease domain of the capsid protein. Cleavage occurs between tryptophan 267 and serine 268 of the polyprotein and releases the capsid protein. After the autoproteolytic cleavage, the free carboxylic group of W267 interacts with the catalytic triad (H145, D167 and S219) and thus leads to self inactivation. Even though the capsid protease is a chymotrypsin like protease, there is an important structural difference to this protease family. The viral protease lacks the C-terminal α-helix present in chymotrypsin and instead forms two short β-strands. These β-strands lead to bending of the C-terminus towards the centre of the capsid protein that allows the last W267 side-chain to reach the active site for auto cleavage to occur [5].

The capsid protease of alphaviruses is well studied. The proteolytic cleavage has been shown to be critical for the life cycle of alphaviruses. A change of S219 to arginine in a conserved tetrapeptide of the catalytic triad, G-D-S(219)-G, of SFV completely abolished *in vitro *cleavage of the capsid protease [7]. All alphaviruses analyzed to date show this autocatalytical cleavage between tryptophan and serine residues. In the SFV capsid, substitution of W267 for three other residues, A, F and R [8] showed that the wild-type W is a better substrate than F for the autoprotease, and that only the W residue allowed efficient viral growth. *In vitro *studies have shown that C-terminal deletion variants of SFV capsid protease and the mutant W267A catalyzed the hydrolysis of ester derivatives of aromatic amino acids (aa) [9].

Most studies on the alphaviral capsid protease have been performed with SFV. We have analyzed for the first time the sequence requirements for the cleavage activity of ChikV capsid protease of genus alphavirus. We also show further evidence about the role of tryptophan 261 in ChikV. Autoproteolytic cleavage of the ChikV capsid is abolished completely upon substituting tryptophan with alanine. Through error-prone PCR we found that isoleucine 227 is also important for the efficient activity of the protease.

## Results

**Figure 1:**
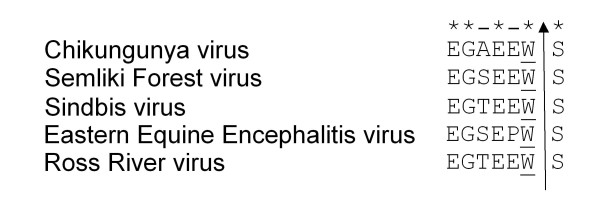
**Conserved amino acids near the cleavage site**. Conserved amino acids near the cleavage site between tryptophan and serine at the C-terminus of the capsid protein of alphaviruses. The arrow indicates the cleavage site.

**Figure 2:**
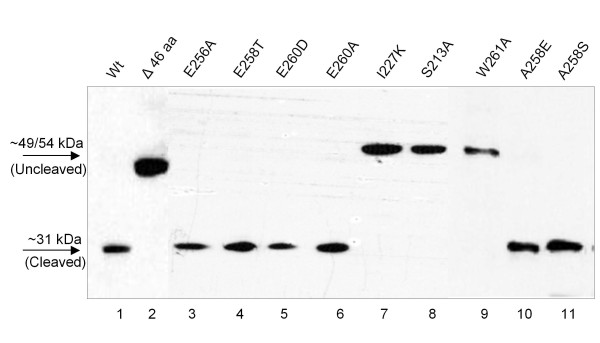
**Mutations affecting the protease activity**. Mutations affecting the protease activity of the Chikungunya virus capsid protease. HEK293 cells were transfected with Capsid-GFP fusion constructs.

Alphaviral replication depends on the efficient and precise cleavage between the tryptophan and serine residue executed by the capsid protease [7]. This cleavage site is conserved in all alphaviruses analyzed including ChikV (Figure 1). In order to determine the sequence requirements for the ChikV capsid protease activity, we cloned the ChikV capsid in fusion with GFP. Figure 2 shows that the ChikV capsid protease efficiently cleaved the capsid from the GFP tag, which also shows that S262 is not important for the cleavage. A mutation of S213 to alanine inactivated the protease and thus prevented cleavage of GFP from the capsid protease (Figure 2, lane 8). Since S213 is an essential part of the autocatalytic triad, this cleavage was executed by the protease domain of the ChikV capsid protease. Thus, the capsid protease activity monitored in our assay depended on the integrity of the catalytic triad of the protease.

We next analyzed if a deletion at the C-terminus retained its protease activity. As shown in Figure 2, lane 2, a deletion of 46 aa from the C-terminus completely abolished the protease activity. While E256, G257 and E259 in ChikV capsid are conserved among alphavirus capsid, the aa in positions 258 and 260 seem to vary specifically between virus species (Figure 1). Crystal structures of SFV and Sindbis virus also show the importance of aa in the C-terminus to anchor the tryptophan residue into the active site [4,5]. Thus, we set out to analyze the influence of the C-terminal aa sequence for the activity of the ChikV capsid protease. As expected, mutating tryptophan in the 261 position to alanine (W261A) completely inactivated the protease (Figure 2, lane 9). We then analyzed if aa near tryptophan in the cleavage site of the protease domain will have any effect on enzyme activity. As shown in the Western blot (Figure 2, lanes 3-6 and 10-11), mutations at these sites were not found to have any effect on the protease activity. Even the substitution of E256 conserved in all alphaviruses to alanine did not have any effect on the activity of the capsid protease.

**Figure 3:**
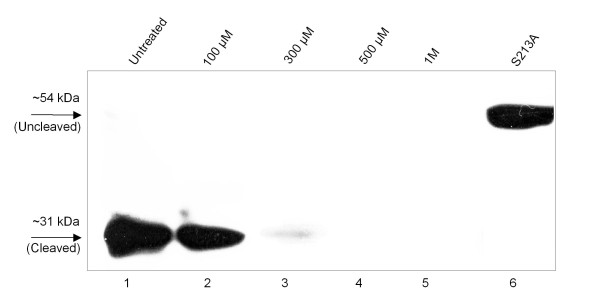
**Effect of AEBSF on protease activity**. Varying concentrations of protease inhibitor AEBSF were added to HEK293 cells transfected with Chikcap-GFP plasmid.

To check if serine protease inhibitor AEBSF (4-(2-Aminoethyl) benzenesulfonyl fluoride hydrochloride) has any effect on the activity of ChikV capsid protease, the inhibitor was used at a concentration of 100 μM to 1 M. AEBSF was not effective in abrogating the cleavage (Figure 3). At higher concentrations, it also inhibited the protein expression (Figure 3, lanes 3-5), mostly due to cytotoxicity.

To identify additional motifs important for the protease activity, we performed an error-prone PCR to introduce mutations. We obtained and analyzed mutants with substitutions of P61L, Q86R, A207V, or I227K. Mutants P61L, Q86R, and A207V of the capsid did not have any effect on the activity of the protease (data not shown). However, an exchange of isoleucine 227 to lysine completely inactivated the protease activity (Figure 2, lane 7).

**Figure 4:**
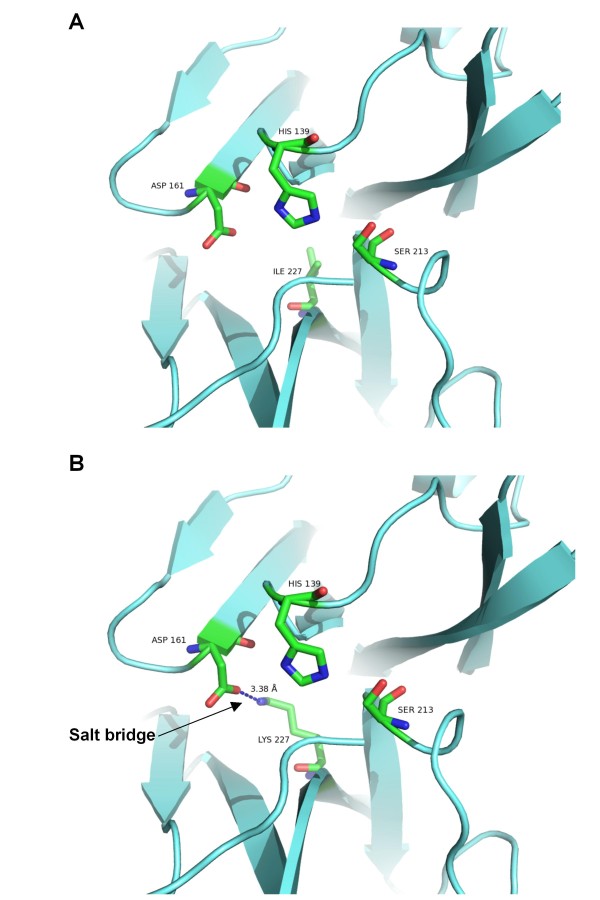
**Molecular modelling of the catalytic triad of ChikV capsid protease**. (A) Wt catalytic triad in native enzyme with isoleucine at 227. (B) The catalytic triad in mutant enzyme with lysine at 227. The side chain of lysine 227 is very close to side chains of the catalytic triad that will disrupt the electrostatic interactions of catalytic triad. The distance between OD1 of aspartic acid 161 and NZ of lysine 227 is 3.38 Å, which is the optimal distance to form a salt bridge between these residues.

So far, the importance of I227 for the activity of alphaviral capsid proteases has not been reported before. Thus, we further analyzed the loss of activity by molecular modelling and comparison of WT and mutant structures. Figure 4 indicates that the lysine introduced at position 227 is spatially very close to the catalytic triad and may disrupt electrostatic interactions in the catalytic site. Rotamers of lysine 227 can make a salt-salt bridge with aspartic acid 161. This rotamer is a preferred conformation of lysine 227 considering the electrostatic interaction with surrounding residues and the absence of steric hindrances (Figure 4B). The 3.38 Å distance between the side chain nitrogen atom NZ of lysine 227 and the side chain oxygen atom OD1 of ASP 161 is the ideal length of a salt bridge which is less than 4 Å. This distance between OD1 and NZ is almost the same before (3.38 Å) and after energy minimization (3.49 Å). Here PDB nomenclature is used in denoting the side chain atoms of aa. NZ is the nitrogen atom of amino group of side chain of lysine. The OD1 is one of the oxygen atoms present in the carboxyl group of the side chain within the aspartic acid.

## Discussion

The cleavage specificity of alphaviral capsid proteases has been characterized partially for SFV and Sindbis virus [4,7]. The main determinants identified for site-specific cleavage is the conservation of W267 (W261 in ChikV) [8] and an intact catalytic triad [7]. An exchange of threonine for serine (S262T) failed to prevent this cleavage, and showed that serine is not important for its activity. Deletion of 46 aa from C-terminus inactivated the protease either due to the missing stretch in the cleavage site or a major change in the tertiary structure. These results also showed that aa within the C-terminus of the ChikV capsid are important for the activity of the protease.

It has been shown before in the SFV capsid protease that only W267 at the cleavage site allowed efficient viral growth [8]. Tryptophan is the preferred amino acid for cleavage as mutating tryptophan in position 261 to the hydrophobic alanine resulted in un-cleaved protein (Figure 2, lane 9).

In the crystal structure of SFV and Sindbis virus capsid W is anchored in the active site, but the C-terminal folding and stability is also dependent on aa 264 to 266, which form an antiparallel β-structure with aa 235 to 237 of the protein [4,5]. Thus, it is possible that aa close to the cleavage site anchor the W to the active site. This prompted us to look for the importance of other aa near to tryptophan. Mutation of other conserved or less conserved residues (Figure 1) close to tryptophan did not alter the activity of the ChikV capsid protease (Figure 2). This further confirms that the prevention of cleavage when substituting tryptophan to alanine is because of an amino acid preference rather than a change in tertiary structure or improper folding, as proposed before [8].

As ChikV capsid encodes a serine protease, we checked if the serine protease inhibitor AEBSF would affect its activity. It was found that AEBSF didn't inhibit the activity even at high concentrations (Figure 3). AEBSF's mode of action is by covalently modifying serine residues in the active sites of proteases such as trypsin, chymotrypsin, plasmin, kallikrein and thrombin. It is also able to modify serine residues on other proteins adding an amine group and thereby, shifting the pI to higher values [10]. Here, lack of inhibition may be a specific property of the alphavirus capsid protease, were it gets self-inactivated after the cleavage.

One mutant introduced by error-prone PCR resulted in complete loss of protease activity when isoleucine at position 227 was changed to lysine. Molecular modelling showed that the formation of a salt bridge between mutant K227 and D161 potentially affects crucial functions of D161 (Figure 4B). D161 is one out of three conserved aa, forming the autocatalytic triad of the capsid protease. The probable salt bridge between K227 and D161 obviously disrupts the protease activity of the autocatalytic triad. In the native enzyme, D161 makes a low barrier hydrogen bond (LBHB) with H139 [11]. Due to this LBHB, H139 acts as general base and abstracts the proton from the S213 hydroxyl group. De-protonated S213 will act as a nucleophile and will form a tetrahedral transition state with carbonyl carbon of the substrate. The disruption of LBHB by N-methylation of histidine [12] or by mutation of D161 of the catalytic triad to asparagine has been shown to inactivate serine proteases [13]. The potential salt bridge between D161 and mutant K227 may also disrupt the LBHB between D161 and H139 and may consequently make the enzyme inactive as the salt bridge can be very stable in the hydrophobic environment of the active site [14].

## Conclusions

In conclusion, our analysis shows that tryptophan, but not the serine near the cleavage site is important for the ChikV protease activity. We also found that I227 is located close to the catalytic triad of the ChikV capsid and a mutation of this residue leads to disruption of the protease activity. Thus we are showing that aa other than the catalytic triads are important for the activity of this serine protease within the ChikV capsid.

## Methods

### Cloning and mutagenesis

Chikungunya capsid protein (1 to 261) was cloned as fusion protein with FLAG tag (DYKDDDDK) at the N-terminus and GFP tag at the C-terminus in pEGFP-N1 vector (Clontech) using primers Flagchik cap_fwd and ChikcapXmaI_WT_rev (Table 1). The PCR products were cloned between the HindIII and XmaI sites of the vector pEGFP-NI (Clontech). All clones were verified by sequencing in both forward and reverse direction. Deletion mutant Δ46 was constructed using primers FlagChik cap_fwd and ChikcapΔ46_rev (Table 1). Other mutations were incorporated by adding mutations in the reverse primer at the C-terminal of the protein (Table 1, 4-10). As template for PCR, plasmid pSMART LCKan containing ChikV structural genes [15] or pChikcap (GenBank: HM369441.1) containing ChikV capsid gene was used.

**Table 1:** Primers used to mutate the capsid protease of Chikungunya virus

Mutation	Primer	Nucleotide sequence (5'-3')
Wt	Flagchik cap_fwd	aagcttatggactacaaagacgatgacgataagaccatggagttcatccc
EGAEEW (wt)	ChikcapXmaI_WT_rev	atcccgggtccactcttcggctc
Δ46 AA (deletes aa 216-261)	ChikcapΔ46_rev	atcccgggttctaccgctgtcccc
W261A (EGAEE**A)**	ChikcapW-A_rev	atcccgggt**cgc**ctcttcggccc (4)
A258E (EG**E**EEW)	ChikcapA-E_rev	atcccgggtccactcttc**ttc**cccctcgg (5)
A258 S (EG**S**EEW)	Chikcap3A-Sr_rev	atcccgggtccactcttc**gga**cccctcgg (6)
E256A (**A**GAEEW)	Chikcap_E-A_rev	atcccgggtccactcttcggcccc**ggc **(7)
A258T (EG**T**EEW)	ChikcapA-Tr_rev	atcccgggtccactcttc**ggt**cccctcgg (8)
E260 D (EGAE**D**W)	Chikcap_E-D_rev	atcccgggtcca**atc**ttcggccc (9)
E260A (EGAE**A**W)	Chik_cap_E-A_rev	atcccgggtcca**cgc**ttcggccc (10)

Serine 213 in the catalytic triad was mutated to alanine by fusion PCR [16] using primers [5'-atggactacaaagacgatgacgataagatggagttcatcccaac-3', 5'-aagatcggtctac**cgg**cgtcccctggttt-3'] and [5'-aaaccaggggac**gcc**ggtagaccgatctt-3', 5'-atcccgggtccactcttcggctc-3']. Error-prone PCR was performed using oligos FlagChik cap and ChikcapXmaI_WT (Table 1) as primers. Reaction mixtures contained 10× PCR buffer (Qiagen), 5 mM Mg^2+^, 10 ng of template DNA, 10 pM of each primer, and 2.5 U of Taq polymerase. The reaction conditions were as follows: step 1, 5 min, 95°C; step 2, 30 s, 94°C; step 3, 30 s, 58°C; step 4, 45 s, 72°C; and step 5, 7 min, 72°C; steps 2 to 4 were repeated 35 times.

### Cell culture, transfection, and inhibition studies

HEK293 cells were maintained in Dulbecco's modified Eagle's medium (DMEM) supplemented with 10% fetal calf serum (FCS), 100 U/ml penicillin, and 100 μg/ml streptomycin. For transfection, HEK293 cells were seeded in 12-well plates at a density of 2 × 10^5 ^cells and transfection was performed using turbofect (Fermentas) transfection reagent according to the manufacturer's instruction. For inhibition studies, serine protease inhibitor AEBSF (4-(2-Aminoethyl) benzenesulfonyl fluoride hydrochloride); (Sigma-Aldrich) at a concentration from 100 μM to 1 M was used.

### Western blotting

48 hours post transfection, cells were washed once with PBS followed by lysis using RIPA buffer (Sigma). Protein samples were subjected to 12% SDS-PAGE and transferred to PVDF membrane (Amersham) by using Xcell II blot module (Invitrogen) and blocked overnight at 4°C with 3% BSA in PBST. The membranes were probed with monoclonal anti-FLAG antibodies (Sigma) followed by anti-mouse secondary antibodies conjugated with horseradish peroxidase (Santacruz). Blots were visualized with the enhanced chemiluminescence (ECL) detection reagents (Amersham, UK).

### Protein modelling and analysis

The structure of the capsid protease was modelled using automated homology modelling server 3D-JIGSAW http://bmm.cancerresearchuk.org/~3djigsaw/[17]. The template, Semliki Forest virus capsid protein (PDB ID 1VCP) is 91% identical in sequence to the ChikV capsid protease domain, which makes the model more reliable. Comparison of WT and mutant structures were done using SPDBV software [18]. The figures were generated using pymol http://www.pymol.org. Energy computations were done with the GROMOS90 implementation of Swiss-PdbViewer.

## Competing interests

The authors declare that they have no competing interests.

## Authors' contributions

ST did the cloning, mutations, expression analysis and protein modelling. JR did the protein models analysis including energy computations. LJ produced some of the clones. SG and CD coordinated with the study and provided study materials. BP provided critical review of the manuscript. SS participated in the study design and wrote the manuscript along with ST. All authors read and approved the manuscript.
